# Attitudes toward E-Cigarettes, Reasons for Initiating E-Cigarette Use, and Changes in Smoking Behavior after Initiation: A Pilot Longitudinal Study of Regular Cigarette Smokers

**DOI:** 10.4236/ojpm.2014.410089

**Published:** 2014-10

**Authors:** Carla J. Berg, Dana Boyd Barr, Erin Stratton, Cam Escoffery, Michelle Kegler

**Affiliations:** 1 Department of Behavioral Sciences and Health Education, Rollins School of Public Health, Emory University, Atlanta, USA; 2 Department of Environmental Health, Rollins School of Public Health, Emory University, Atlanta, USA

**Keywords:** E-Cigarettes, Cessation, Harm Reduction, Longitudinal Study, Nicotine Biomarkers

## Abstract

**Objectives:**

We examined 1) changes in smoking and vaping behavior and associated cotinine levels and health status among regular smokers who were first-time e-cigarette purchasers and 2) attitudes, intentions, and restrictions regarding e-cigarettes.

**Methods:**

We conducted a pilot longitudinal study with assessments of the aforementioned factors and salivary cotinine at weeks 0, 4, and 8. Eligibility criteria included being ≥18 years old, smoking ≥25 of the last 30 days, smoking ≥5 cigarettes per day (cpd), smoking regularly ≥1 year, and not having started using e-cigarettes. Of 72 individuals screened, 40 consented, 36 completed the baseline survey, and 83.3% and 72.2% were retained at weeks 4 and 8, respectively.

**Results:**

Participants reduced cigarette consumption from baseline to week 4 and 8 (p’s < 0.001); 23.1% reported no cigarette use in the past month at week 8. There was no significant decrease in cotinine from baseline to week 4 or 8 (p’s = ns). At week 8, the majority reported improved health (65.4%), reduced smoker’s cough (57.7%), and improved sense of smell (53.8%) and taste (50.0%). The majority believed that e-cigarettes versus regular cigarettes have fewer health risks (97.2%) and that e-cigarettes have been shown to help smokers quit (80.6%) and reduce cigarette consumption (97.2%). In addition, the majority intended to use e-cigarettes as a complete replacement for regular cigarettes (69.4%) and reported no restriction on e-cigarette use in the home (63.9%) or car (80.6%).

**Conclusions:**

Future research is needed to document the long-term impact on smoking behavior and health among cigarette smokers who initiate use of e-cigarettes.

## 1. Introduction

Electronic cigarettes (or e-cigarettes) are battery-powered devices that contain liquid that is vaporized with inhalation of the e-cigarette without any combustion or smoke and consist of a combination of propylene glycol, vegetable glycerine, various levels of nicotine, and flavor concentrates [1]. There is a great variability in the size, shape, appearance, and nicotine content of e-cigarettes [2]. The emergence of e-cigarettes in the US may enable smoking cessation for some cigarette smokers because it addresses the biochemical and behavioral aspects of smoking addiction [3], the latter of which is largely unaddressed with traditional nicotine replacement therapies (NRT). However, there is research needed in order to establish the effect of e-cigarettes on the smoking behavior of cigarette smokers in order to inform the regulation of e-cigarettes and their marketing.

A number of studies have demonstrated the efficacy of e-cigarettes in alleviating cravings for cigarettes [4] [5]. Moreover, recent studies have examined how e-cigarettes are being used and their potential as a harm reduction or cessation aid. One study followed 40 current regular smokers (*i.e*., smoking 15 cigarettes per day [cpd]) experimenting with e-cigarettes for 24 weeks (27 retained at follow-up) [6]. They reported a six-month point prevalence smoking cessation rate of 22.5%, with an additional 32.5% of smokers reducing their cigarette consumption by at least 50% [6]. A cross-sectional survey of 222 first-time purchasers of e-cigarettes conducted in 2010 [7] found that the 6-month point prevalence of smoking abstinence among the e-cigarette users in the sample was 31.0%. Roughly two-thirds reported a reduction in cpd and almost half reported abstinence from smoking for a period of time. Of respondents who were not smoking at 6 months, 34.3% were not using e-cigarettes or any nicotine-containing products at the time. The largest longitudinal study to date included 1329 e-cigarette users (367 retained after one year) over a one-year period [8]. This study documented a 31% sixmonth point prevalence abstinence. Among dual users (smokers who were vaping daily at baseline), 22% had stopped smoking after one month, and 46% had stopped after one year. In dual users who were still smoking at follow-up, cigarette consumption decreased by on average 5.3 cpd after one month, but remained unchanged between baseline and one-year follow-up. Each of these longitudinal studies of e-cigarette users demonstrated high rates of attrition.

Only two randomized controlled trials (RCTs) to date have examined the effects of e-cigarette use on smoking cessation. One study of 657 smokers motivated to quit in New Zealand who were randomized to nicotine e-cigarettes, patches, and placebo e-cigarettes found that, at 6 months, verified abstinence was higher (7.3%) among those using nicotine e-cigarettes versus those using patches (5.8%) and those using placebo e-cigarettes (4.1%); they also found greater reduction in cpd in the e-cigarette group than the patches group [9]. Another RCT of smokers unmotivated to quit found that they substantially reduced cig/day use from baseline by more than 50% in both participants provided nicotine-containing e-cigarettes and those not containing nicotine, and reductions in cpd were unrelated to the nicotine content in the cartridges [10]. These findings suggest the promise of e-cigarettes in potentially assisting in achieving cessation.

Sources of information regarding e-cigarettes, attitudes about e-cigarettes, and reasons for use have received limited attention in research. Regarding sources of information, one study [11] found that television was the most common source of information, followed by in-person conversations, and internet sources. In terms of reasons for use, a cross-sectional study of 3587 e-cigarette users [12] found that reasons for using e-cigarettes included the perception that it was less toxic than tobacco (84%), to deal with craving for tobacco (79%) and withdrawal symptoms (67%), to quit smoking or avoid relapsing (77%), because it was cheaper than smoking (57%) and to deal with situations where smoking was prohibited (39%). Other information regarding these phenomena or restrictions related to e-cigarettes versus regular cigarettes in different environments is lacking. Another study of cigarette smokers who also used e-cigarettes [13] found that the most common reason for using e-cigarettes was to reduce consumption of regular cigarettes, with 60% reporting that electronic cigarettes helped them to achieve this.

There is controversy regarding e-cigarettes and how e-cigarettes should be regulated. Proponents of e-cigarettes indicate that e-cigarettes may result in harm reduction, whereas opponents argue that too little is known about the health impact of e-cigarettes and effectiveness of e-cigarettes in supporting cessation, among other concerns [14]. Given the aforementioned research and the gaps in examination of regular smokers’ first-time use of e-cigarettes over time, the current pilot study recruited regular smokers who purchased e-cigarettes for the first time at an e-cigarette store and 1) examined changes in smoking and vaping behavior, associated cotinine levels, and health status from baseline to weeks 4 and 8 and 2) assessed their attitudes, intentions, and restrictions regarding e-cigarettes and e-cigarette use. This is one of the first naturalistic observation studies examining regular cigarette smokers recruited through an e-cigarette (vape) shop as they made their first e-cigarette purchase and experimented with e-cigarettes for the first time. This research will inform a subsequent naturalistic observational study of first-time e-cigarette purchasers or users.

## 2. Methods

### 2.1. Participants & Procedures

The current research protocol was reviewed and approved by the Emory University Institutional Review Board. This study took place in Atlanta, where there were no official policies or restrictions regarding e-cigarette use during this study. Participants were recruited from a community partner, an e-cigarette vendor who has two stores in the metro-Atlanta area and that exclusively sells e-cigarettes that have a cartomizer storing e-cigarette liquid in a reservoir, along with a wide range of liquids in terms of nicotine dose and flavors. Participants were recruited through fliers posted in the store.

Eligibility criteria included being ≥18 years old, smoking ≥25 of the last 30 days, smoking ≥5 cpd, smoking regularly for ≥1 year, and not having started using e-cigarettes. Interested participants obtained a study packet from the e-cigarette vendor staff (who volunteered to distribute packets) and then called the research staff while in the e-cigarette store and were screened for eligibility. Eligible participants were instructed to complete the baseline study assessment and mail it to the research office using the pre-addressed stamped envelope. The study packet included two consent forms, one of which they returned completed along with the assessment and one of which they kept for their records.

The sample of 36 participants was recruited from July 2013 to September 2013. Of 72 people screened for eligibility, 42 were eligible. Of the 42 eligible, 36 completed and returned the baseline assessment. Retention rates at week 4 and week 8 were 83.3% (n = 30) and 72.2% (n = 26), respectively. Our sample size of completers at weeks 4 and 8 (n = 30 and n = 26) provided us with 80% power to detect an average of 3 cpd and 4 cpd change in cpd from baseline to each follow-up, respectively. There were no significant differences between those retained and those lost to follow-up.

### 2.2. Measures

Participants completed mail-based, pencil-and-paper survey assessments at baseline, 4 weeks, and 8 weeks, receiving a $75 Amazon gift card for each assessment completed. Each follow-up assessment (conducted at week 4 and week 8) was mailed to participants, and participants received up to six reminder calls and/or text messages to notify them that the assessments were due.

#### Sociodemographics

Participants reported their age, gender, race/ethnicity, employment status, and education level [15].

#### Smoking Characteristics

At each assessment, participants reported the number of days they smoked in the past 30 days, average cpd, whether they mainly used menthol cigarettes, and brand of cigarettes typically used [15]. They also completed a 7-day timeline follow-back indicating the number of cigarettes smoked on each day of the past 7 days [16].

Participants reported the level of importance of and confidence in quitting on a 10-point scale [17]. Participants were also asked, “During the past 12 months, how many times have you stopped smoking for one day or longer because you were trying to quit smoking?” [18]. This question was adapted to assess number of quit attempts in the past four weeks at each follow-up assessment. Participants were also asked, “Which of the following have you used to help you quit smoking? (Check all that apply.): I have never tried to quit smoking; I quit on my own, did not use anything; Talked to a doctor or nurse for help with quitting; Talked to a counselor; Attended a class or group program; Used telephone counseling; or Used an Internet or online program; Nicotine patch; Nicotine gum; Nicotine lozenge; Zyban, Wellbutrin, or Bupropion; or Champix or Varenicline [19].”

#### E-Cigarette Characteristics

At baseline, participants responded to a range of newly developed assessments to examine this emerging behavior for which there are few (if any) standardized behavioral assessments. Participants were asked if they purchased tobacco flavored liquid for their e-cigarettes. We also asked participants, “What strength of e-cigarette liquid did you obtain?” Response options included: “0 mg/ml; 6 mg/ml; 12 mg/ml; 16 mg/ml; 18 mg/ml; 26 mg/ml; or I don’t know [12].” At weeks 4 and 8, they were asked to indicate the number of days in the past 30 they used their e-cigarette and average number of times per day they used it [12]. They were also asked to complete a 7-day timeline follow-back indicating the number of times they used their e-cigarette on each day of the past 7 days [16].

#### Biomarker Assessment

At each assessment, saliva samples were collected by active saliva induction and collection using Salivette (Sarstedt, Newton, NC) kits. Participants were provided saliva collection kits and instructions in the assessment packets and then mailed the samples and the surveys to study staff. Cotinine was analyzed by the lab at Emory University for cotinine by liquid chromatography-atmospheric pressure chemical ionization tandem mass spectrometry (LC-MS/MS) as described by Jacob *et al*. [20]. The limit of detection of the method was 0.1 ng/ml with an average relative standard deviation on quality control samples of 8% and an average RSD of duplicate analyses of 18%. The linear range of the method was 0.1 – 200 ng/ml. Samples >200 ng/ml were diluted and repeated if the requisite sample was available. For those samples with cotinine >200 ng/ml without enough sample to repeat the analysis, this value (200 ng/ml) was imputed for data analyses. For those samples with cotinine >1000 ng/ml, this value (1000 ng/ml) was imputed for data analyses.

#### Assessments of Health Change

At weeks 4 and 8, participants were asked to indicate a response of “Better, The Same, Or Worse” to each of the statements listed in Table 1 [21].

#### Sources of Information about E-Cigarettes

Participants were asked, “How did you first learn about e-cigarettes?” Response options included: “Television news stories; News stories in newspapers and magazines; Advertisements in newspapers and magazines; Online advertisements; From friends and family; From my doctor or nurse; or Other [11].” Participants were also asked, “Have you ever talked to your doctor or nurse about using e-cigarettes to quit smoking?” Response options included: “No; Yes, and they recommended that I try it; Yes, but they didn’t know enough about them; or Yes, and they recommended against using them.”

#### Attitudes toward E-Cigarettes

Participants were asked to include their level of agreement (1 = *Strongly disagree* to 5 = *Strongly agree*) with statements listed in Table 2, which were newly developed for this study. Response options were categorized in *agree* and *strongly agree* vs. *neutral* vs. *disagree* and *strongly disagree*.

#### Intentions for E-Cigarette Use

Participants responded to a newly developed assessment asking, “How do you plan to use the e-cigarettes? (Check all that apply.) [21].” Response options are listed in Table 2.

#### Smoking and Vaping Restrictions

In terms of personal spaces, we asked participants to report whether they allowed smoking or vaping in their home or car, respectively, using and adapting other nationally and internationally used assessments [15] [22]. Participants were asked, “Which statement best describes the rules about smoking regular cigarettes [or vaping e-cigarettes] inside your home [or inside your car]?” Response options included: “Smoking [or vaping] is not allowed anywhere inside your home [or car” (*i.e.*, complete restriction); “Smoking [or vaping] is allowed in some places or at some times” (*i.e.*, partial restriction); or “Smoking [or vaping] is allowed anywhere inside the home [or car” (*i.e*., no restriction). We also asked, “If you are employed, is smoking regular cigarettes allowed at your worksite?” Response options included: “Yes, No, or I am not employed.” This question was adapted to ask about rules related to e-cigarettes.

#### Open-Ended Questions at Week 4 Assessment

In order to capture retrospective reports of intentions for e-cigarette use and experiences of initial e-cigarette use, participants were asked a number of open-ended questions at week 4. These questions are listed in Table 3.

### 2.3. Data Analysis

Participant characteristics were summarized using descriptive statistics, and paired samples t-tests were conducted to examine differences from baseline to week 4 and to week 8. We also examined the impact of regular cigarette smoking and e-cigarette use in the past seven days on cotinine levels at week 4 and week 8 using ordinary least squares regression. Specifically, we entered average regular cpd and number of times of use of e-cigarettes per the seven-day timeline follow-back assessments of each product. All statistics were conducted using SPSS 21.0, and alpha was set at 0.05. For the open-ended questions, responses were reviewed and categorized by major themes (e.g., type of information source; positive vs. negative vs. neutral; social group), and representative quotes from responses were selected to qualitatively describe select phenomena and presented in Table 3.

## 3. Results

The study sample was an average age of 36.06 (SD = 15.28) years, with 55.6% (n = 22) being female, and 88.9% (n = 32) being White. In addition, 19.4% (n = 7) were unemployed, 58.4% (n = 21) had some college education, 19.5% (n = 7) earned at least a Bachelor’s degree.

### 3.1. Smoking and Cessation History

Baseline measures of smoking characteristics (see Table 1), indicated that participants smoked an average of 29.78 (SD = 0.96) days of the past 30 and an average of 14.85 (SD = 6.05) cpd. Additionally, 28.5% (n = 10) were menthol smokers, and 63.9% (n = 23) were Marlboro smokers. In terms of prior cessation attempts, 61.1% (n = 22) had not made a quit attempt in the past 12 months, with 16.7% (n = 6) in total reporting never trying to quit in their lifetime. Of the 30 participants that had tried to quit smoking in their lifetime, 46.7% (n = 14) reported attempting to quit without the use of any resources or support; 50.0% (n = 15) used the nicotine patch; 30.0% (n = 9) used nicotine gum; 20.0% (n = 6) used nicotine lozenges; 36.7% (n = 11) used Zyban, Wellbutrin, or Bupropion; 23.3% (n = 7) used Chantix or Varenicline; 6.7% (n = 2) talked to a healthcare provider; 10.0% (n = 3) talked to a counselor; 13.3% (n = 4) used a group program; and 3.3% (n = 1) used telephone counseling.

### 3.2. Smoking and Vaping Behavior

Regarding the e-cigarette liquid, 58.3% (n = 21) of participants purchased a tobacco-flavored liquid. Regarding nicotine strength, 11.1% (n = 4) purchased 12 mg/ml, 25.0% (n = 9) purchased 16 mg/ml, 30.6% (n = 11) purchased 18 mg/ml, and the remainder could not remember.

Table 1 shows a reduction in overall cigarette consumption from baseline to weeks 4 and 8 as assessed by number of days of the past 30 smoked, average cpd in the past 30, and average cpd in the past 7 days (per the 7-day timeline follow-back; p’s < 0.001, respectively). Compared to baseline cpd per the 7-day timeline follow-back, 93.3% (n = 28/30) reported lower cigarette consumption at week 4 (average decrease = 9.33, SD = 7.21) and 92.3% (n = 24/26) reported lower cigarette consumption at week 8 (average decrease = 9.61, SD = 7.79). In addition, 23.1% (n = 6) reported no cigarette use in the past 30 days or 7 days at week 8. Conservatively, assuming that those lost to follow-up continued smoking cigarettes, 30-day point prevalence abstinence from regular cigarettes was 8.3% and 16.7% at weeks 4 and 8, respectively, and 7-day point prevalence abstinence from regular cigarettes was 22.2% and 16.7% at weeks 4 and 8, respectively. Moreover, participants reduced the average number of times using their e-cigarettes per day in the past 7 week 4 to week 8 (p < 0.001).

There was no significant decrease in cotinine from baseline to week 4 (p = 0.389) or to week 8 (p = 0.387). In the regression examining cotinine levels at week 4, neither average cpd in the past 7 days per the 7-day timeline follow-back (Beta = 1.06, 95% Confidence Interval [CI] −16.30, 18.42, p = 0.901) nor the average times using the e-cigarette (Beta = 1.76, CI −1.50, 5.01, p = 0.278) predicted cotinine levels (R-squared = 0.052). However, in the regression model examining cotinine levels at week 8, average cpd for the past 7 days predicted cotinine levels (Beta = 14.47, CI 1.35, 27.59, p = 0.032), but average times using the e-cigarette did not (Beta = 1.26, CI −3.17, 5.69, p = 0.558; R-squared = 0.219). In terms of change in health symptoms, at week 8, the majority of participants reported a range of health benefits. There was also no significant difference in average cotinine levels between those who reported 30-day point prevalence abstinence at week 8 versus those who continued to smoke (abstinent M = 113.97, SD = 66.75 vs. not abstinent M = 214.58, SD = 208.99, p = 0.266).

### 3.3. Attitudes, Intentions, and Restrictions Related to E-Cigarettes Use

Most participants (88.9%; n = 32) had heard about e-cigarettes from friends and family, while the remainder (11.1%, n = 4) heard about them from TV news stories or advertisements (see also Table 3). In addition, 74.3% (n = 26) had not asked their doctor about using e-cigarettes to quit smoking. Of the 25.7% (n = 9) who had asked their healthcare provider about them, 8.6% (n = 3) said their doctor recommended that they try it, and 17.1% (n = 6) reported that their doctor said that they did not know enough about them (see also Table 3).

Table 2 presents data regarding attitudes and knowledge about e-cigarettes, their intent in using e-cigarettes, and their personal restrictions regarding smoking versus vaping and the related restrictions at work. The majority believed that e-cigarettes have fewer health risks than regular cigarettes (97.2%), that e-cigarettes should be allowed where smoking is not (88.9%), and that e-cigarettes have been shown to help smokers quit (80.6%) and to reduce cigarette consumption (97.2%). Only 27.8% believed that the Food and Drug Administration (FDA) should regulate e-cigarettes.

Most reported that they intended to use e-cigarettes as a complete replacement for regular cigarettes (69.4%). Fewer participants reported that they were planning to use e-cigarettes where they cannot use regular cigarettes (25.0%) or that they were just experimenting (8.3%) or trying something different (11.1%; see also Table 3).

In terms of restrictions regarding vaping in the home and in the car, most (63.9% and 80.6%, respectively) reported no restriction on e-cigarette use. In addition, 61.1% reported that vaping was allowed at work (vs. 33.3% reporting that smoking was allowed).

### 3.4. Reactions to Using E-Cigarettes

Table 3 provides results of the open-ended questions included at week 4, including the questions, some major themes that emerged, and sample responses representing the themes. We also quantified the number of participants who responded to the open-ended question asking if they had talked to anyone about their use of e-cigarettes, if they had allowed others to use their e-cigarette, and if they would recommend e-cigarettes to others. These data indicated that 86.4% (n = 19/22) said that they had, mostly to friends, family, or healthcare providers. In addition, 54.5% (n = 12/22) reported that they had allowed others, mostly friends, to use their e-cigarette. In total, 90.9% (n = 20/22) said that they would recommend e-cigarettes to others.

## 4. Discussion

This study is one of the first to examine the experiences of regular smokers as they initiate use of e-cigarettes. We found that 23.1% reported no cigarette use in the past 30 days at week 8, and the vast majority reported lower cigarette consumption at follow-up. Moreover, participants reduced their average number of times per day using their e-cigarettes from week 4 to 8. These findings add to the body of literature indicating that e-cigarettes may be useful in reducing cigarette consumption among regular smokers who initiate use [6]–[8] [23]. In addition, most participants reported some health benefit of using e-cigarettes, particularly improvements in overall health, reduced smoker’s cough, and increased sense of taste and smell.

It is important to note that there were no significant decreases in cotinine from baseline to week 4 or 8. This is not surprising, given that e-cigarettes contain nicotine and result in escalated cotinine levels [24]–[27]. Moreover, levels of e-cigarette use may vary over time or increase initially due to compensating for nicotine cravings [26]. In addition, at week 8, average cpd for the past 7 days predicted cotinine levels, but average times using the e-cigarette did not. Prior research has found that substituting tobacco cigarettes with e-cigarettes may substantially reduce exposure to selected tobacco-specific toxicants [28]. Given these findings and prior literature, more research is needed to understand the association between e-cigarette use patterns and cotinine levels over time and when e-cigarettes are concurrently used with other tobacco products in order to inform the regulation of e-cigarettes.

We also found that the vast majority of participants had heard about e-cigarettes from friends and family. In addition, the majority had not asked their doctor about using e-cigarettes to quit smoking. Of those who had asked their healthcare provider about them, one-third said their doctor recommended that they try it, and the other two-thirds reported that their doctor said that they did not know enough about them. Given the limited empirical evidence of the effectiveness of e-cigarettes in promoting or supporting cessation, more research is needed immediately to inform both consumers and healthcare providers. Moreover, the overwhelming majority of participants believed that e-cigarettes have fewer health risks than regular cigarettes, should be allowed where smoking is not, and have been shown to help smokers quit and to reduce cigarette consumption. Interestingly, only 27.8% believed that the FDA should regulate e-cigarettes. This may be related to relatively little understanding of the role of the FDA in protecting and informing consumers. Alternatively, these consumers may generally reject high levels of regulation and assume that regulation will impact access to e-cigarettes. Further research is needed to understand consumer perceptions of the FDA and its role.

Most participants reported that they intended to use e-cigarettes as a complete replacement for regular cigarettes, and a quarter of participants reported that they were planning to use e-cigarettes where they cannot use regular cigarettes or that they were just trying something different. This is consistent with a previous study [12] that found that smokers’ reasons for using the e-cigarette included the perception that it was less toxic than tobacco, to deal with craving for tobacco and withdrawal symptoms, to quit smoking or avoid relapsing, to save money, and to deal with situations where smoking was prohibited.

Furthermore, participants generally reported positive experiences with e-cigarettes in comparison to regular cigarettes and to other cessation resources. Participants reported believing that e-cigarettes are healthier for the user and those around them, that they satisfy the behavioral component of smoking, and that they address withdrawal symptoms, which aligns with previous findings [13] [29].

Regarding restrictions about e-cigarette use in personal spaces and at work, most reported no restriction of use. In addition, most reported that vaping was allowed at work, while fewer reported that smoking was allowed. In contrast, many participants reported restrictions applying to cigarette smoking in home, in the car, and at work. The rates of smoking restrictions are promising; however, additional information is needed to understand the true impact of e-cigarette use among those exposed to secondhand vapor, as prior research indicates that using an e-cigarette indoor may involuntarily expose nonusers to nicotine but not to toxic tobacco-specific combustion products [30] [31].

The majority of participants used liquids with nicotine concentrations between 12 mg/ml and 18 mg/ml at all three time points, which is similar to the average of 16 mg/ml found by Etter and colleagues [26]. In addition, the vast majority of participants in the current study continued using the e-cigarette by week 8. This is in line with prior research [8] [13] indicating that almost all daily vapers continue to vape over time. These findings have important implications for research and practice. Future research should examine cessation and harm reduction outcomes among current smokers using e-cigarettes in a larger sample and across a longer period of time. In order to determine the population-level impact of e-cigarettes, research is needed to explore the risk and trajectories of uptake of these products among both smokers and nonsmokers [23]. Varied methodological approaches are also needed to examine the emerging phenomenon of alternative nicotine delivery devices among users and those around them in general. In terms of practice, policy makers must craft policies, regulations, recommendations, and educational campaigns that address the changing context of nicotine consumption and account for the pioneering literature regarding e-cigarettes.

### Limitations

This study has some limitations. First, this study was a small pilot study on a small budget that prohibited more elaborate data collection. As such, the small sample size restricts our power. In addition, our weeks 4 and 8 retention rates were 83.3% and 72.2%, respectively. Those lost to attrition may have been more likely to have stopped using the e-cigarette or to have continued smoking regular cigarettes. There may also be bias inherent in recruitment through a local e-cigarette vendor, especially given the variety of e-cigarette products on the market. We also did not assess type of e-cigarette purchased; however, our recruitment site—the e-cigarette vendor— indicated that the majority of first-time purchasers of e-cigarettes at this shop purchase the refillable tank style. We also cannot ensure that participants completed the baseline assessments prior to initiating e-cigarette use. However, we recorded the number of days between initial contact to verify eligibility and receipt of the assessment; all packets were received in the mail within 8 days. In addition, the sample was drawn from individuals living in one southeastern US state, further limiting its generalizability. Furthermore, future research might benefit from a longer follow-up period and additional biomarker assessments (e.g., NNAL). On a related note, exposure to secondhand smoke was not assessed and could not be accounted for in the analyses examining predictors of salivary cotinine.

## 5. Conclusion

The current study documented significant decreases in cigarette consumption from baseline to week 4 and to week 8 as assessed by all measures. In addition, 23.1% reported no cigarette use in the past 30 days at week 8. Moreover, participants reduced their use of e-cigarettes from weeks 4 to 8. Most participants intended to use the e-cigarette as a total replacement for regular cigarettes and had highly favorable experiences with the e-cigarettes in comparison to regular cigarettes compared to other cessation aids. Finally, we documented very low rates of personal rules restricting where e-cigarettes are used, suggesting that additional research is needed to examine the impact of secondhand vapor on those around e-cigarette users.

## Figures and Tables

**Table 1:** Participant smoking and vaping characteristics and changes in health at baseline, week 4, and week 8.

Variable	Baseline n = 36	Week 4 n = 30	p-value	Week 8 n = 26	p-value
*Smoking Characteristics*				
No smoking, past 30 days (%)	-	3 (10.0)^c^	-	6 (23.1)^d^	-
Number days smoking, past 30 days (SD)	29.77 (0.96)	17.45 (12.84)	<0.001	16.88 (13.01)	<0.001
Ave cpd, past 30 days (SD)	14.85 (6.05)	6.01 (5.71)	<0.001	6.36 (6.15)	<0.001
No smoking, past 7 days (%)	-	8 (26.7)^e^	-	6 (23.1)^f^	-
Ave cpd, past 7 days (SD)	14.57 (6.10)	5.23 (5.91)	<0.001	5.99 (6.41)	<0.001
*Vaping Characteristics*				
No vaping, past 30 days (%)	36 (100.0)	-	-	1 (4.0)	-
Number of days vaping, past 30 days (SD)^a^	-	27.74 (4.79)	-	24.56 (9.20)	0.108
Ave e-cig uses, past 30 days (SD)^a^	-	21.81 (28.68)	-	13.00 (17.64)	0.615
No vaping, past 7 days (%)	36 (100.00)	-	-	1 (4.0)	-
Ave e-cig uses, past 7 days (SD)^a^	-	23.83 (31.63)		11.75 (17.97)	<0.001
Cotinine (SD)	230.58 (223.13)	200.86 (232.19)	0.389	188.34 (186.59)	0.387
*Psychological Characteristics*					
Importance (SD)	8.76 (1.64)	8.59 (2.09)	0.620	8.46 (2.56)	0.172
Confidence (SD)	6.52 (2.95)	7.55 (2.44)	0.108	7.08 (2.50)	0.331
Quit attempt, past 12 months (%)	14 (38.9)	-	-	-	-
Quit attempt, past 4 weeks (%)	-	23 (76.7)	-	15 (57.7)	-
Number quit attempts, past 4 weeks (SD)^a^	-	2.46 (4.30)	-	2.57 (4.62)	0.674
*Symptoms, N (%) indicating “better” rather than “same”*					
Since starting to use the e-cigarette, do you feel your health is:	-	15 (50.0)	-	17 (65.4)	-
If you had a smoker’s cough before using the e-cigarette, is it now:	-	15 (50.0)	-	15 (57.7)	-
How has your ability to exercise changed since using the e-cigarette?	-	9 (30.0)	-	12 (46.2)	-
How has your sense of smell changed since using the e-cigarette?^b^	-	16 (53.3)	-	14 (53.8)	-
How has your sense of taste changed since using the e-cigarette?	-	12 (40.0)	-	13 (50.0)	-

Note: All comparisons done from baseline to week 4 and from baseline to week 8.

a Exceptions: Comparison from 4-week to 8-week assessment;

b 1 participant indicated worse. Note: Assuming those lost to follow-up continued use of cigarettes,

c 8.3%,

d 16.7%,

e 22.2%, and

f 16.7% were abstinent, respectively.

**Table 2:** Baseline attitudes toward e-cigarettes, intentions for use, and smoke- and vape-free restrictions among new e-cigarette initiators who are current smokers, n = 36.

Variable	n (%)
*Attitudes toward E-Cigarettes*^a^	
E-cigarettes have fewer health risks in comparison to regular cigarettes	35 (97.2)
You should be able to use e-cigarettes in places that do not allow smoking	32 (88.9)
E-cigarettes have been shown to help smokers quit	29 (80.6)
E-cigarettes help people cut down on cigarettes or quit smoking	35 (97.2)
The FDA should regulate e-cigarettes^b^	10 (27.8)
*Intentions for Use*^c^	
I plan to use them when I cannot use cigarettes	9 (25.0)
I plan to use them in addition to regular cigarettes	1 (2.8)
I plan to use them as a partial replacement for regular cigarettes	6 (16.7)
I plan to use them as a complete replacement for regular cigarettes	25 (69.4)
I plan to gradually switch from regular cigarettes to e-cigarettes	14 (38.9)
I plan to eventually quit the use of e-cigarettes and regular cigarettes	17 (47.2)
I plan to just experiment with it—I have not yet made up my mind	3 (8.3)
I just wanted to try something different and new	4 (11.1)
*Rules about Smoking and Vaping*	
Home smoking restriction^c^	
Completely smoke-free	28 (77.8)
Partial	4 (11.1)
No	4 (11.1)
Car smoking restriction^c^	
Completely smoke-free	10 (27.8)
Partial	9 (25.0)
No	17 (47.2)
Home vaping restriction^c^	
Completely vape-free	2 (5.6)
Partial	11 (30.6)
No	23 (63.9)
Car vaping restriction^c^	
Completely vape-free	0 (0.0)
Partial	7 (19.4)
No	29 (80.6)
Smoking allowed at work^d^	12 (33.3)
Vaping allowed at work^d^	22 (61.1)

a Indicated *strongly agree* or *agree* versus *neutral*, *disagree*, or *strongly disagree*;

b All responses other than *strongly agree* or *agree* were *neutral*, with the exception of 19.4% (n = 7) that *disagreed* or *strongly disagreed* with “the FDA should regulate e-cigarettes”;

c Indicated *yes* versus *no*;

d 19.4% (n = 7) were not employed.

**Table 3:** Open-ended questions, themes, and sample responses representative of the major themes. Concept and Question Themes and Sample Responses

Concept and Question	Themes and Sample Responses
*Sources of Information about E-Cigarettes*	
1) “Where did you first hear about e-cigarettes and what did you hear about them?”	*Friends* “I was with a friend at a bar. She asked if I wanted to try it because we were talking about quitting. She’s quit for over a year and only uses them at bars.” *Family* “My sister, she said they were great.” *Store/Retail* “In convenient stores. I didn’t hear anything except they don’t stink.”
2) “Have you ever talked to your doctor or nurse about e-cigarettes? If you did, what did you ask them? What did they say?”	*Yes*/*Recommendation* “Yes, I asked what they knew about safety. They did not know much, but agreed it was worth trying. Very supportive.” *Yes*/*No Recommendation* “Yes but they did not know much about them.” *No* “No I have not talked to my doctor about them yet.”
*Experience Using E-Cigarettes*	
1) “What made you decide to try the e-cigarette? How did you actually use it? Did you use it as recommended? When did you plan to use it?”	*Cessation Aid* “Quit smoking regular cigarettes.” *Healthier*; *No SHS* “For health reason and the smell of regular cigarettes. Also [I want to reduce] second hand smoke around family.” *Expense* “A friend uses them, and in the long run they save money. The flavors are appealing, and [e-cigarettes] don’t pollute.”
2) “Tell me about the first time you tried an e-cigarette.”	*With Friends*; *Talking about Quitting Smoking* “I was with a friend at a bar. She asked if I wanted to try it because we were talking about quitting.” *Experiment with Other*’*s E-Cigarettes* “I was at work, about ready to leave. My coworker let me try hers, so I could compare the taste to a regular cigarette.” *Experimented Alone* “I was alone in my car driving. To try and quit smoking.”
3) “How much did you overlap use between the regular cigarettes and the e-cigarette?”	*Frequently* “A lot. Something the e-cig wasn’t really sufficient for my craving so I would smoke a regular cigarette in between usage some days.” *Sometimes* “Off and on for a week or so. I have cut back regular cigarettes by more than half since starting the e-cigarette, but not completely quit.” *Never*/*Almost Never* “I quit cigarettes all together when starting the e-cigarette. I had a few cigarettes since, but did not enjoy them.”
4) “How effective was it for addressing your withdrawal symptoms? Your cravings? The hand to mouth movement? Other triggers?”	*Not Very* “It does seem to help, but am having a more difficult time. It is particularly difficult around smokers.” *Somewhat* “It’s good for most cravings—extreme emotional triggers are harder.” *Very* “Very effective. No withdrawal at all.”
5) “At what point did you think you might quit smoking regular cigarettes completely? Did this ever happen? Do you think you will?”	*No Plan* “I don’t know at what point. It hasn’t happened. I don’t think I want to quit.” If I do, it will probably be winter time.” *Yes*/*Quit in Future* “I’m working on it, someday I will [quit regular cigarettes].” *Yes*/*Quit Immediately/Soon* “Over the next 10 days, I’ll supplement wit e-cigarettes and dropping the amount of regular cigarettes each day until I can say ‘that’s it’.”
6) “What are the advantages of e-cigarettes over regular cigarettes?”	*Smell*; *Impact on Others*; *Impact on Health* “Less odor, less offensive to non-smokers.” I don’t inhale as deeply so less cough.” *Impact on Health*; *Impact on Others* “No tar, carbon dioxide, or other chemicals that regular cigarettes have. It is far more socially acceptable.” *Impact on Health* “Health has improved; improved taste/smell. I have saved money.”
7) “What are the disadvantages of e-cigarettes over regular cigarettes?”	*Inconvenience* “None other than bulkier and heavier.” “Cravings for a regular cigarette.” *Concerns about Unknown* “I have no idea what sort of chemicals are in the juice which raises some red flags. I can’t compare e-cigarettes vs. regular cigarettes.” *May Use More*/*Fewer Restrictions* “I tend to smoke it a lot more in one sitting than I would just one real cigarette.”
8) “How does the e-cigarette compare to other resources or products you have used for quitting smoking?”	*Address Habit*; *Reduce Nicotine over Time* “It is by far more promising. It combines the ritual, nicotine, and perceived ‘smoke’. It will allow me to slowly titrate.” *Address Habit*; *Flavors* “The imitation (hand to mouth), blowing out vapor, and variety of flavors and is superior to other nicotine supplements.” *Address Habit* “I had better results with nicotine lozenges. I’m thinking about trying those again. The e-cigarettes reinforce the oral habit which might be a bad thing.” *Address Cravings* “I’m still getting nicotine in an e-cigarette. I prefer an inhibitor to craving like Chantix but couldn’t handle the side effects. E-cigarettes are much safer.”
1) “Have you talked about e-cigarettes with anyone in the past month? What was said? Did anyone else try your e-cigarette? Why? What did they say?”	*No* “No, not really.” *Yes*/*Received Support* “I have talked to family and friends; they were very supportive.” *Yes*/*Endorse to Others* “Yep. I just talk about what I know. It’s cheaper, lacks carcinogens, and you can smoke it indoors.”
2) “Would you recommend it to others to reduce smoking or to quit smoking? Why or why not?”	*Yes*/*Health* “Yeah! I feel better in more ways than one and I don’t smell gross!” *Yes*/*No SHS* “Yes. Because it is better for others around who do not smoke.” *Unsure* “I’m not far enough along yet to recommend them for quitting but would for smoking where prohibited.”
